# A Case Report of Management of Medulloepithelioma of the Ciliary Body and Iris without Recurrence over an Observation Period of Twenty Years

**DOI:** 10.1155/2023/1508341

**Published:** 2023-01-27

**Authors:** N. Eide, J. Navaratnam, P. Jebsen

**Affiliations:** ^1^Department of Ophthalmology, Oslo University Hospital, Oslo, Norway; ^2^Department of Pathology, Oslo University Hospital, Oslo, Norway

## Abstract

Intraocular medulloepithelioma is a rare embryonal tumor that is believed to arise from the epithelium of the medullary tube. We report a 37-year-old female with medulloepithelioma presented at the age of 17 with a one-month history of left-sided visual deterioration and visible iris lesion. Birth history and medical and family histories were insignificant. The left eye revealed a vascularized iris mass. Further examination revealed a grey-white ciliary body mass and a subluxated lens with best-corrected visual acuity (BCVA) of 0.5. The patient underwent partial lamellar corneo-sclerouvectomy. The histological and electron microscopic findings revealed medulloepithelioma. To reduce the risk of recurrence of the probable malignant tumor, she was treated with Ruthenium plaque therapy about six weeks following surgical removal. Pars plana vitrectomy and lensectomy with laser photocoagulation of the peripheral retina were performed at the removal of the brachytherapy plaque. She regained her BCVA of 1.0 in her left eye 3.5 months following pars plana vitrectomy. At 20-year follow-up, no tumor recurrence was seen and her BCVA remained 1.0.

## 1. Introduction

Intraocular medulloepitheliomas arise from the primary medullary epithelium along the inner layer of the optic cup. Intraocularly, it commonly arises from the ciliary body epithelium but may also arise from the iris, retina, and the optic nerve head. Verhoeff published a histological description of the tumor and named it teratoneuroma [1]. Grinker termed the tumor medulloepithelioma due to the resemblance of the tumor to the neuroepithelium of the embryonic neural tube [2]. The tumor is commonly diagnosed in patients' at the first decade of life [3, 4]. The most common presenting symptoms of medulloepithelioma include visual deterioration, pain, mass in the iris or ciliary body, and leukocoria [4]. The frequent clinical findings include cyst or mass in the iris, anterior chamber, or ciliary body; elevated intraocular pressure; glaucoma; and cataract [ 4]. Here, we report a case of medulloepithelioma presenting with unilateral visual deterioration, mass on the iris, and cataract.

## 2. Case Presentation

The patient presented at the age of 17 with a one-month history of a whitish lesion on the iris and diminished vision in her left eye. The ophthalmological examination revealed a polypous, vascularized iris mass from the 5 to 7 o'clock position and a fleshy, grey-white ciliary body tumor that was partially pigmented on the surface. Diascleral transillumination proved to be negative. Lens subluxation with opacities was seen. The tumor size measured 6.8 × 8.2 × 5.2 mm on ultrasonography. The intern reflectivity was high with variable homogeneity and without the presence of cysts.

The examination of her right eye was within normal limits. The BCVA was 1.0 o. dexter and 0.5 o. sinister, and intraocular pressure was 13 mmHg o. dexter and 14 mmHg o. sinister. Her visual fields on Donders' test were also normal.

**Figure 1:**
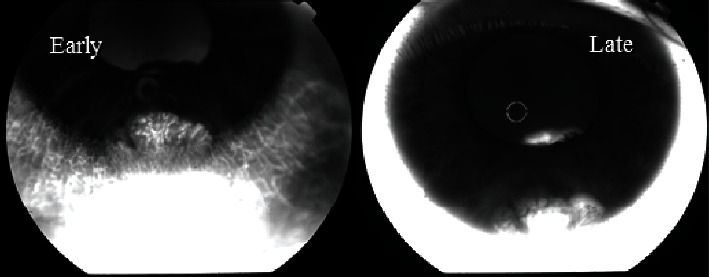
Fluorescein angiography demonstrating hyperfluorescence in the early and late phases of the tumor on the iris from the 5 to 7 o'clock position and ciliary body (Zeiss). Part of the tumor is visible in the pupillary area posterior to the iris in the late-phase image.

The fluorescein angiography demonstrated a hyperfluorescent tumor (Figure 1). Radiological investigations including chest X-ray, ultrasonography of the liver and abdomen, and computed tomography of the orbit and brain did not reveal any pathological lesions. An iris-ciliary body melanoma was one of the tentative diagnoses. Therefore, bone marrow (BM) aspiration was performed from the iliac crest as a part of our ongoing study of micrometastasis [5]. The cells collected were examined with a monoclonal antimelanoma antibody, 9.2.27, which binds to an epitope on the surface of melanoma cells. Remarkably, the test was positive and negative one year after the excision of her tumor.

**Figure 2:**
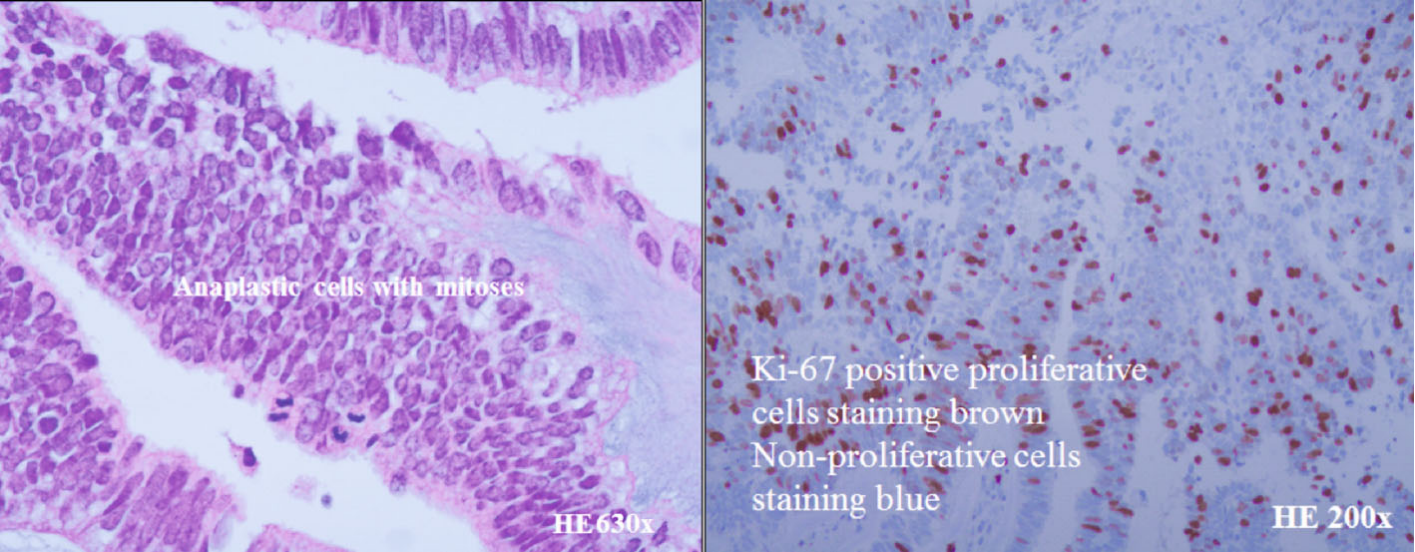
Anaplastic cells with mitosis and Ki-67-positive proliferative cells are shown. The cells are stained positive for S-100, CD56, and factor VIII (not shown in these figures). The rapid growth indicated by positive Ki-67, a proliferating marker, and the presence of anaplastic cells were the main reasons for the diagnosis of malignant medulloepithelioma. HE(S): hematoxylin-eosin (saffron).

Surgical removal of the tumor was performed with partial lamellar corneo-sclerouvectomy. Cord-tubular structures without rosettes of the tumor epithelium, amorphous fibrilla of the tumor stroma, nuclear atypia, and mitosis were demonstrated histologically (Figure 2). The cells stained positive for S-100, Ki-67, CD56, and factor VIII and negative for PanMel, anti-M-actin, and cytokeratin (Figure 2). Histological and electron microscopic findings were consistent with a medulloepithelioma. The specimen was sent abroad for evaluation. The diagnosis was confirmed, and enucleation was recommended.

**Figure 3:**
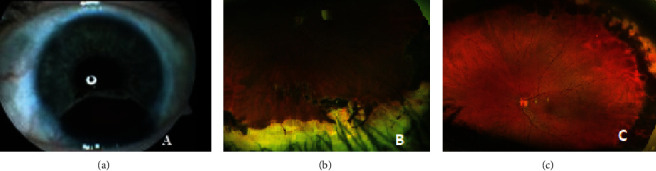
(a, b) Iris coloboma of the left eye following surgical treatment for medulloepithelioma (a) (Zeiss Clarius 700) and status following resection of the tumor (b) and laser-photocoagulated retina in the periphery (c) (Optos California) are shown. These images are taken 20 years following surgical removal of the malignant medulloepithelioma.

She underwent Ruthenium plaque therapy about six weeks following surgical removal of the tumor due to risk of tumor recurrence (dose of 60 Gy on the apex, dose rate of 0.8 Gy/hour, and scleral dose of 325 Gy). At the removal of brachytherapy plaque, the patient underwent pars plana vitrectomy with laser photocoagulation of the peripheral retina at 360 degrees. The patient regained her BCVA of 1.0 at the 3.5-month follow-up following pars plana vitrectomy. Thirteen years following surgical treatment of the tumor, her intraocular pressure increased to 28 mmHg in her left eye, and treatment with topical beta-blockers once a day was started and is still continued. The patient developed dizziness a year before the diagnosis of glaucoma during her first of two pregnancies, and a diagnosis of multiple sclerosis was made two years following the diagnosis of glaucoma. There were no eye symptoms. The diagnosis was supported with magnetic resonance imaging and polyclonal bands in the cerebrospinal fluid. She underwent treatment initially with teriflunomide and later with cladribine. She does not have any multiple sclerosis symptoms with normal activity and had not been on any treatment for the past two years. However, if she tries to accelerate the tempo, the deficient left limb coordination limited such activities. Her BCVA remained at 1.0 with stable refraction in both eyes and without tumor recurrence at 20-year follow-up. Optic discs are normal without signs of glaucoma or optic atrophy. The intraocular pressure remains within normal limits with topical beta-blockers. The only restriction is an advice to avoid contact sport. An iris coloboma between 4 and 7 o'clock, thin sclera, an aphakic left eye with laser-photocoagulated peripheral retina were evident on examination (Figures 3(a) and 3(b)). The resection combined with irradiation can be an eye-salvaging treatment in medulloepithelioma of the ciliary body and iris.

## 3. Discussion

The medulloepithelioma is usually diagnosed in the first decade of life [3, 4]. Our patient was 17 years old at the time of presentation, in good health, and with a one-month presenting history. Therefore, differential diagnosis was more in focus. Although malign melanoma is more common in Scandinavia, her tumor did not have the typical appearance of malign melanoma. This aspect was discussed in depth with the patient and her parents. We proposed the finding to be a malignant tumor. Due to her age of presentation, lack of clinical features such as pain, neovascular glaucoma, and retrolental membrane, and rarity of medulloepithelioma, the accurate diagnosis could not be made clinically. The patient and her parents strongly preferred eye-saving treatment modalities, although we were sceptical to the final visual outcome. Therefore, partial lamellar corneo-sclerouvectomy was performed. Generally, biopsy of tumor in the eyes or intravitreous/intracameral access has been discouraged due to the concern of tumor dissemination. Since the modified technique of intravitreous injection in eyes with retinoblastoma was reported by Munier et al. in 2012, protocols have been developed for intravitreous injection of chemotherapy for the treatment of the tumor in the eye (particularly retinoblastoma) [5]. Our patient underwent eye-saving surgical resection of the tumor due to the patient's and her parents' wishes.

The medulloepithelioma demonstrates primitive neuroepithelial cells arranged as cords that closely resemble the primitive retina. About 20% of medulloepithelioma cases are associated with persistent fetal vasculature or persistent hyperplastic primary vitreous. It can be classified into nonteratoid or teratoid and further subclassified as benign or malignant. The nonteratoid medulloepithelioma consists of cells originating from the primitive medullary epithelial elements [4]. The teratoid medulloepithelioma contains heteroplastic elements of mesenchymal cells, hyaline cartilage, rhabdomyoblasts, or neuroglial cells in addition to cells originating from the medullary epithelial proliferation [4]. We did not detect the teratoid elements in the tumor. However, there could be a small nest. We find a very high number of cells in her BM staining with our melanoma marker in our micrometastatic study [6]. Therefore, we consider the neoplasm as a malignant teratoid medulloepithelioma. The retinoblastoma-like elements with or without rosettes, sarcoma-like elements, pleomorphism, high mitotic index, and invasion into adjacent structures such as the lens, uvea, sclera, cornea, optic nerve, or orbit indicate malignant medulloepithelioma [4]. We perceive the excised tumor to be a malignant tumor. The rapid growth of cells, anaplastic cells, high mitotic number, and positive Ki-67, a proliferating marker, were the fundament for this conclusion. Therefore, we consider that the irradiation with Ruthenium plaque was essential for the exceptional outcome without local recurrence or metastatic disease. We followed her up for five years with routine imaging of the liver.

Smaller medulloepithelioma may be treated with cryotherapy, radiotherapy, or local resection. Shields et al. reported that 6 out of 10 patients treated with local resection required additional treatment due to tumor recurrence [7]. Five of the 6 patients treated with local resection underwent enucleation or exenteration, four due to local recurrence, and one due to ocular inflammation and discomfort. One of the patients was treated with cryotherapy [7]. This tumor type shows highly invasive growth and penetration of the sclera also when not classified as a malignant type. Combining the case series reported by Broughton and Zimmerman (56 eyes with intraocular medulloepithelioma) [4], Canning et al. (16 eyes) [8], and Kaliki et al. (35 eyes) [9], the main primary and secondary treatment modalities consist of enucleation (88 cases), exenteration (10 cases), partial lamellar sclerouvectomy (22 cases), and plaque brachytherapy (5 cases). Subsequent tumor treatment was required in 15 out of 35 cases [9]. Primary enucleation has been the most common treatment modality for ocular medulloepithelioma due to the high tumor recurrence rate [7, 9]. Our patient was treated with local resection of the malignant tumor, subsequent Ruthenium brachytherapy, and pars plana vitrectomy. The combined treatment modalities have been eye-salvaging with a BCVA of 1.0 20 years following treatment.
